# 1283. Native Wrist Septic Arthritis: a 10-year Single Center Experience

**DOI:** 10.1093/ofid/ofad500.1122

**Published:** 2023-11-27

**Authors:** Pansachee Damronglerd, Ryan B Khodadadi, Said El Zein, Jack W McHugh, Gina A Suh, Aaron J Tande, Brandon J Yuan, Omar M Abu Saleh

**Affiliations:** Faculty of Medicine Thammasat University, Rochester, Minnesota; Mayo Clinic, Rochester, Minnesota; Mayo Clinic, Rochester, Minnesota; Mayo Clinic, Rochester, Minnesota; Mayo Clinic, Rochester, Minnesota; Mayo Clinic, Rochester, Minnesota; Mayo Clinic Rochester, Rochester, Minnesota; Mayo Clinic Rochester, Rochester, Minnesota

## Abstract

**Background:**

Native joint septic arthritis (NJSA) of the wrist is a rare condition, with published literature suggesting an incidence of approximately 1.5% among patients presenting with acute painful and swollen wrist. The aim of this study was to investigate the characteristics, management, and outcomes of wrist NJSA at our institution.

**Methods:**

We conducted a retrospective study of all adults diagnosed with wrist NJSA who underwent surgical intervention at Mayo Clinic facilities from January 2012 to December 2021. The diagnosis was confirmed based on clinical presentation, synovial fluid white blood cell count, and aspiration or operative cultures.

**Results:**

A total of 557 patients developed NJSA during the study period, of whom 55 (9.9%) were diagnosed with wrist NJSA, median age was 63, with the associated demographics (**Table 1**). 12 (21.8%) had a history of antecedent trauma or procedure, 11 (20%) had synchronous joint infections, and 22 (40%) had positive blood cultures. The median synovial white blood cell count was 94,050/mm^3^ (IQR 51,920 – 139,941 /mm^3^). Crystals were detected in 5 patients (8.8%). Additional diagnostic data are available in **Table 2**. Open debridement was performed in 35 (61.4%) patients, and the rest underwent arthroscopy. Positive Gram stain results were detected in the preoperative synovial fluid of 12 (21.8%) cases. The most common pathogen isolated was *Staphylococcus aureus* in 13 (24%) of cases. The median duration of antimicrobial therapy was 31 days (IQR 26 – 45 days). There was no statistically significant difference in the incidence of complications between patients who underwent open debridement and those who underwent arthroscopy (**Table 3**). The most common complication of both groups was compromised range of motion of the wrist joint.

**Figure:**
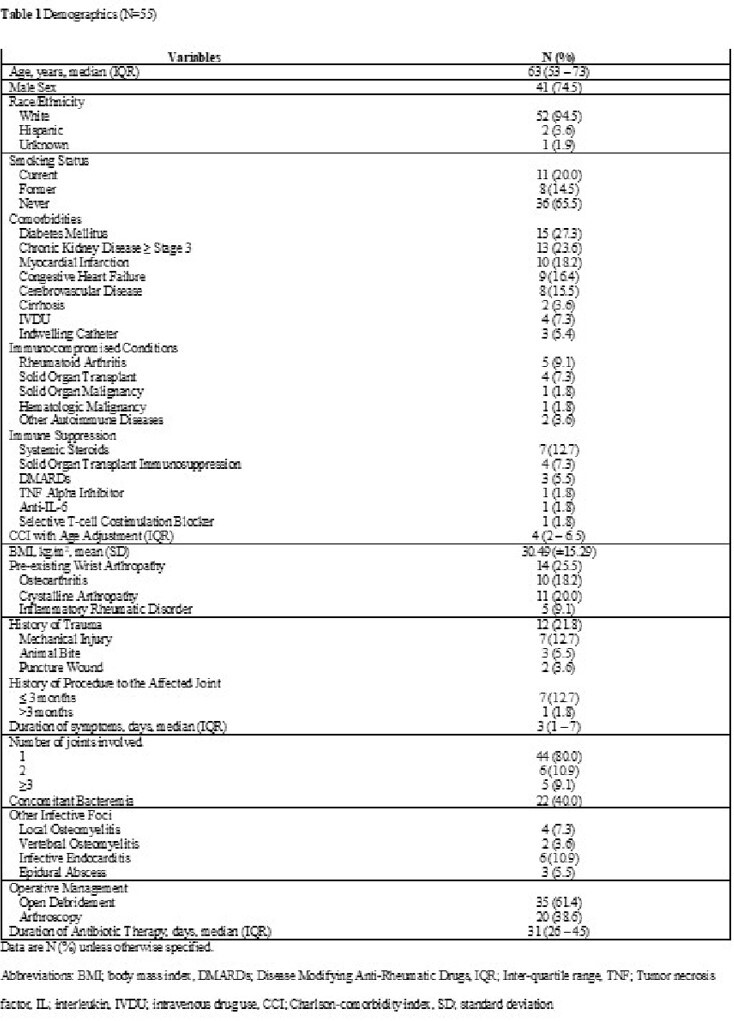

**Figure:**
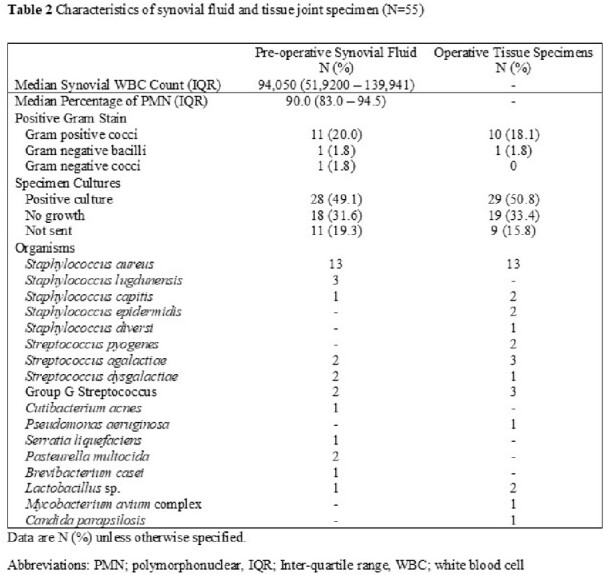

**Figure:**
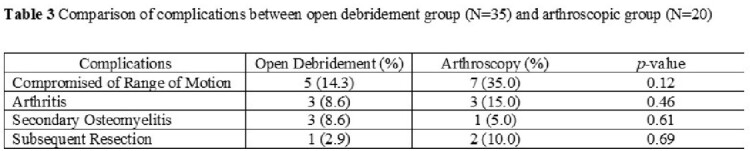

**Conclusion:**

Native wrist septic arthritis comprised 9.9 % of all NJSA during the study period. Concomitant bacteremia and synchronous joint infections were common, highlighting the importance of obtaining blood cultures and comprehensive joint exam in this setting. Crystals and Gram stains were detected in 10 % and 22% of cases, respectively. These findings should not rule out NJSA. Decreased range of motion was common complication, affecting 21% of all cases.

**Disclosures:**

**Gina A. Suh, M.D.**, Adaptive Phage Therapeutics: Grant/Research Support|Adaptive Phage Therapeutics: IP royalties|Phagelux: Grant/Research Support **Aaron J. Tande, M.D.**, Musculoskeletal Infection Society: Board Member|Wolters Kluwer Health - Lippincott Williams & Wilkins: Publishing royalties, financial or material support **Brandon J. Yuan, M.D.**, American Association of Orthopaedic Surgeons: Board Member|DePuy, A Johnson & Johnson Company: Advisor/Consultant|Mid America Orthopaedic Association: Board Member|Stryker: Advisor/Consultant

